# Premature Senescence of T-cells Favors Bone Loss During Osteolytic Diseases. A New Concern in the Osteoimmunology Arena

**DOI:** 10.14336/AD.2021.0110

**Published:** 2021-08-01

**Authors:** Luis González-Osuna, Alfredo Sierra-Cristancho, Carolina Rojas, Emilio A Cafferata, Samanta Melgar-Rodríguez, Angélica M Cárdenas, Rolando Vernal

**Affiliations:** ^1^Periodontal Biology Laboratory, Faculty of Dentistry, Universidad de Chile, Santiago, Chile.; ^2^Faculty of Dentistry, Universidad Andres Bello, Santiago, Chile.; ^3^Department of Periodontology, School of Dentistry, Universidad Científica del Sur, Lima, Perú.; ^4^Health Sciences Division, Faculty of Dentistry, Universidad Santo Tomás, Bucaramanga, Colombia.; ^5^Department of Conservative Dentistry, Faculty of Dentistry, Universidad de Chile, Santiago, Chile.

**Keywords:** senescence, T-lymphocytes, CD28, RANKL, osteoimmunology, bone loss

## Abstract

Cellular senescence is a biological process triggered in response to time-accumulated DNA damage, which prioritizes cell survival over cell function. Particularly, senescent T lymphocytes can be generated prematurely during chronic inflammatory diseases regardless of chronological aging. These senescent T lymphocytes are characterized by the loss of CD28 expression, a co-stimulatory receptor that mediates antigen presentation and effective T-cell activation. An increased number of premature senescent CD4^+^CD28^-^ T lymphocytes has been frequently observed in osteolytic diseases, including rheumatoid arthritis, juvenile idiopathic arthritis, ankylosing spondylitis, osteopenia, osteoporosis, and osteomyelitis. Indeed, CD4^+^CD28^-^ T lymphocytes produce higher levels of osteoclastogenic molecular mediators directly related to pathologic bone loss, such as tumor necrosis factor (TNF)-α, interleukin (IL)-17A, and receptor-activator of nuclear factor κB ligand (RANKL), as compared with regular CD4^+^CD28^+^ T lymphocytes. In addition, premature senescent CD8^+^CD28^-^ T lymphocytes have been negatively associated with bone healing and regeneration by inhibiting osteoblast differentiation and mesenchymal stromal cell survival. Therefore, accumulated evidence supports the role of senescent T lymphocytes in osteoimmunology. Moreover, premature senescence of T-cells seems to be associated with the functional imbalance between the osteolytic T-helper type-17 (Th17) and bone protective T regulatory (Treg) lymphocytes, as well as the phenotypic instability of Treg lymphocytes responsible for its trans-differentiation into RANKL-producing exFoxp3Th17 cells, a key cellular phenomenon directly related to bone loss. Herein, we present a framework for the understanding of the pathogenic characteristics of T lymphocytes with a premature senescent phenotype; and particularly, we revise and discuss their role in the osteoimmunology of osteolytic diseases.

Cellular senescence is defined as a biological process characterized by the arrest of the cell cycle, frequently accompanied by changes in cell phenotype, in response to accumulated DNA damage [1]. Indeed, when cumulative damage to the DNA overwhelms cellular repair mechanisms, cells activate either the program of cell death (apoptosis) or senescence [2]. From an immunological point of view, these processes greatly differ since apoptosis is usually followed by inflammation resolution, while cellular senescence mainly favors inflammation and contributes mostly to immunopathogenic reactions, by prioritizing cell survival instead of cell function [2].

Senescent cells display molecular and morphological changes which translate into heterogeneous functional variations, and these are dependent on the type of affected cell and the type, intensity, and duration of the stimulus that provoked the DNA damage [2, 3]. Even though multiple pathways can lead to cellular senescence and result in equally diverse senescent phenotypes, these pathways seem to converge into a common cell phenotype transversal to the different types of senescent cells (Fig. 1) [2, 3]. After the damage to the genetic material occurs, cells activate the DNA damage response (DDR), which can be detected by the identification of specific markers, such as phosphorylated histone 2AX (γ-H2Ax) [3-5]. In turn, γ-H2Ax positively regulates genes linked to cell cycle arrest, such as p21 and p53, in order to prevent the replication of the defective cell [3-5]. Apart from that, senescent cells accumulate lysosomal content, evidenced by the increase in the activity of senescence-associated-β-galactosidase (SA-βgal), which has been related to lysosomal alterations, autophagy dysfunction, and accumulation of damaged mitochondria [6, 7].

**Figure 1.**
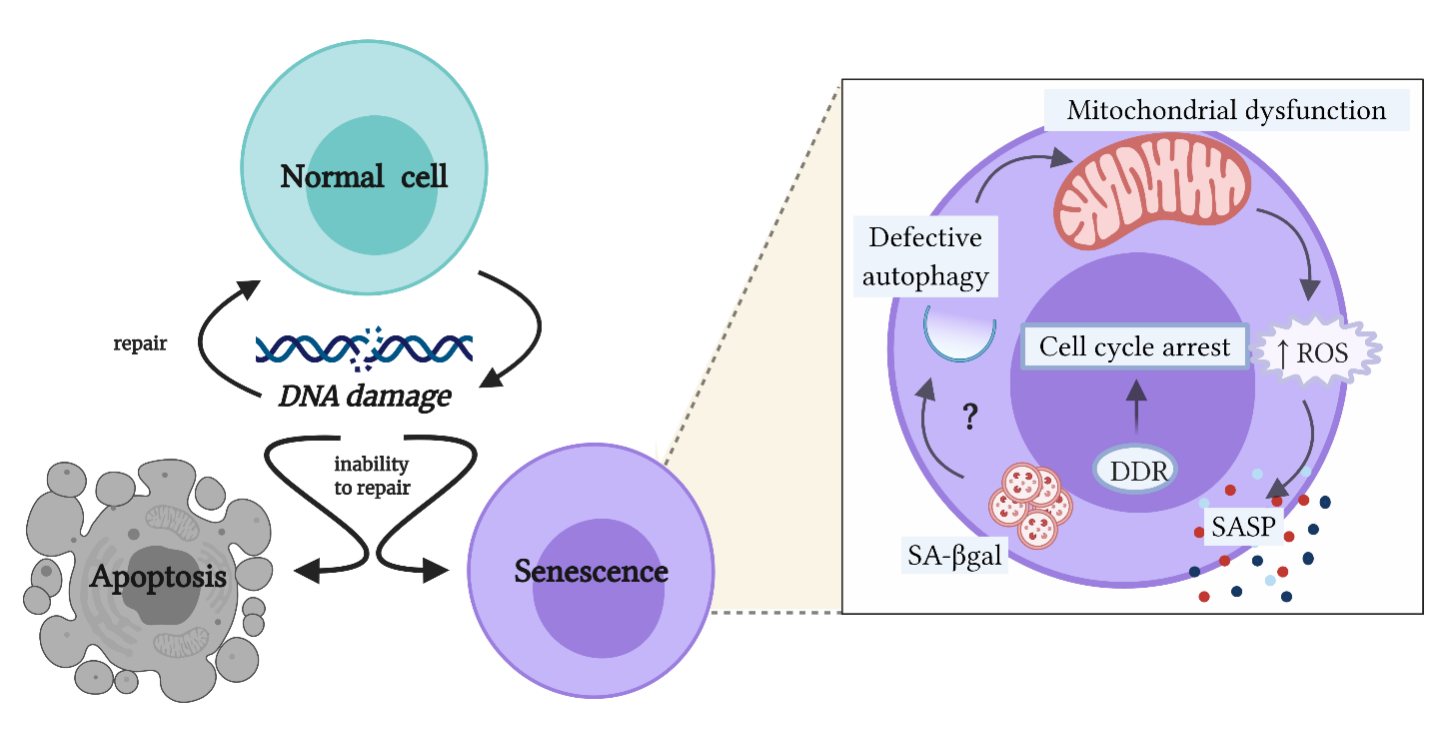
Cellular senescence as an alternative pathway to apoptosis after accumulated DNA damage. When accumulated DNA damage occurs, the affected cells can repair the DNA damage to continue their programmed functions or, when their repair mechanisms cannot restore the integrity of damaged DNA, cells can initiate either the apoptosis program or senescence program. In fact, cell senescence is an alternative fate to apoptosis that prioritizes cell survival instead of cell function. The programming of the common phenotype among senescent cells is initiated by the DNA damage response (DDR) that leads to the arrest of the cell cycle, in order to avoid the replication of the defective cell. Then, cells present an increase in the activity of senescence-associated-β-galactosidase (SA-βgal), which is linked to lysosomal failure and potential autophagy alterations; however, this link remains unclear. In turn, the defective autophagy observed in senescent cells can lead to the accumulation of dysfunctional mitochondria and consequently to the increase of the production of reactive oxygen species (ROS). Besides, these cells can acquire a senescence-associated secretory phenotype (SASP), producing increased levels of cytokines, chemokines, growth factors, and proteases (Created with BioRender.com).

Functionally, senescent cells acquire pro-inflammatory properties, characterized by increased production of reactive oxygen species (ROS), derived from dysfunctional mitochondria, and the establishment of a senescence-associated secretory phenotype (SASP), responsible for the increased production of cytokines, chemokines, growth factors, and proteases [4, 8]. The SASP-related molecular pattern produced by senescent cells depends on the type of affected cell and the senescence-inducing factor [9]. In addition, it contributes to the autocrine maintenance of the senescent phenotype and the induction of senescence in neighboring cells, a process called paracrine senescence or bystander effect [10, 11]. Therefore, SASP and ROS maintain a vicious circle of inflamm-aging, where chronic inflammation leads to senescence, and senescence contributes even more to unceasing inflammation [12].

Notably, there are no actual unique markers for the identification of cellular senescence [13]; however, the absence of CD28 expression has been proposed as a characteristic that could allow the identification of senescent T lymphocytes [14].

## T-cell senescence: CD28^-^ T lymphocytes

During the antigenic presentation, T lymphocytes are activated when their T-cell receptor (TCR) recognizes the antigen/MHC II complex and their CD28 receptor binds to the costimulatory molecules CD80 or CD86, expressed by antigen-presenting cells [15]. The loss of CD28 expression is considered the most consistent biological indicator of cellular senescence in T lymphocytes and the critical aging marker in the human immune system [14]. CD28^-^ T lymphocytes, also called CD28^null^ T lymphocytes, present common characteristics of the senescent cell phenotype, such as the upregulation of γ-H2Ax and p53, and a robust increment in the SA-βgal activity [16-18]. CD28^-^ T lymphocytes are considered dysfunctional, oligoclonal, terminally differentiated, and with limited proliferative capacity, although with an increased capacity to produce pro-inflammatory cytokines [14, 19] and resistance to the suppressive function of T regulatory (Treg) lymphocytes and the effect of steroids [20, 21]. In addition, CD28^-^ T lymphocytes are highly resistant to apoptosis-triggering signals and accumulate in high quantities in the elderly [22, 23].

**Figure 2.**
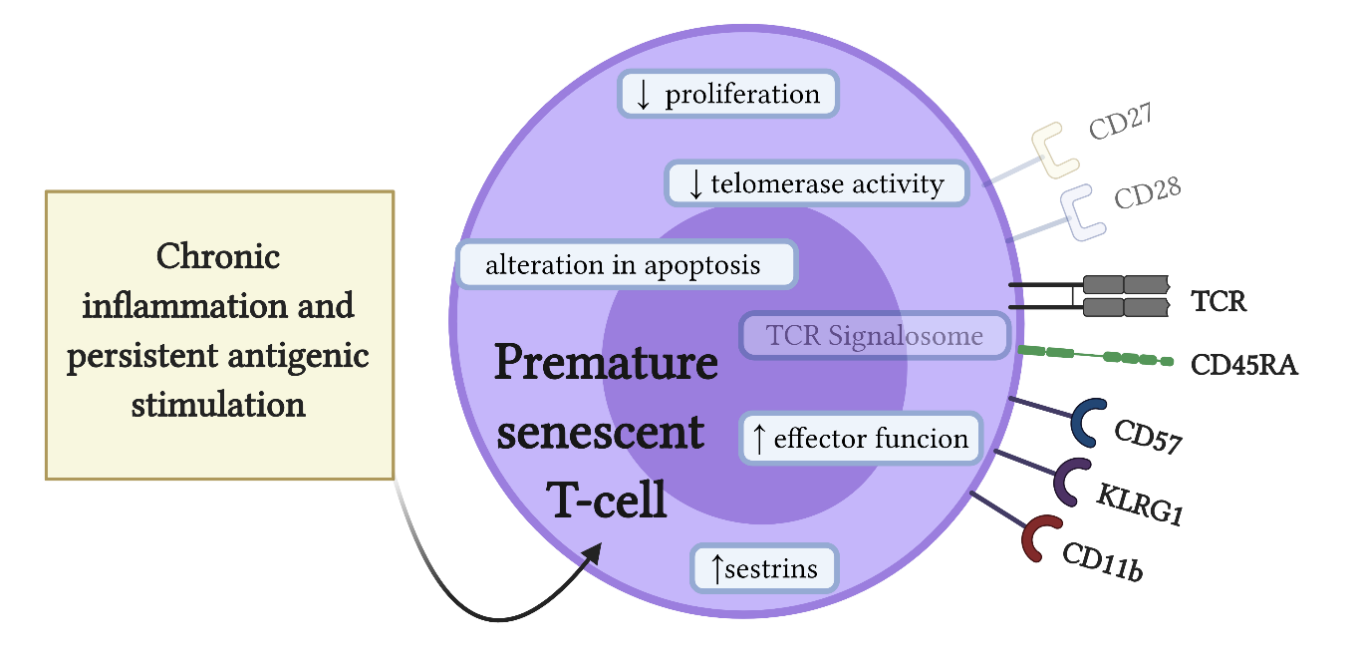
Hallmarks of premature senescent T lymphocytes. Chronic inflammation and persistent antigenic stimulation can induce premature senescence of T cells. Premature senescent T lymphocytes express traditional senescent cell characteristics, such as increased DNA damage response (DDR), increased cell cycle arrest protein levels, such as p53, and senescence-associated-β-galactosidase (SA-βgal) activity. In addition, senescent T lymphocytes express specific cell markers, such as the loss of co-stimulatory receptors CD28 and CD27, as well as the loss of the signalosome molecules Zap70 and Lck, involved in the T-cell receptor (TCR) signaling. Furthermore, premature senescent T lymphocytes have a diminished proliferative capacity, low telomerase activity, and altered apoptosis. However, they maintain potent effector functions, including the expression of innate immunity receptors, such as CD11b and CD57, and the co-inhibitory killer cell lectin-like receptor G1 (KLRG1). Recently, it has been proposed that sestrin proteins could be involved in the development of the T-cell senescence phenotype (Created with BioRender.com).

It is noteworthy that senescent T lymphocytes can also be generated prematurely during chronic diseases regardless of chronological aging [19], since chronic inflammation can lead to immunosenescence, particularly in T lymphocytes [24]. In this sense, T lymphocytes seem to be more susceptible to cellular senescence than other immune cells for several reasons: (1) they are dependent on thymic integrity for cell renewal, (2) they undergo great proliferative stress due to massive clonal expansion during their activation, (3) they have long periods of life as carriers of immune memory, and (4) they are subjected to high oxidative and metabolic stress, which leads to accumulation of unresolved DNA damage [25].

Various pathways can lead to premature senescence of T cells. During repetitive T lymphocyte stimulation involving clonal expansion, the CD28 expression may be irreversibly lost by transcriptional silencing [26]. In addition, the loss of CD28 expression in T lymphocytes is accompanied by a decreased telomerase activity, which can lead to telomere shortening, the reason why T-cell senescence is frequently attributed to a process of replicative senescence [27, 28]. Indeed, somatic cells have a finite number of cell divisions known as the Hayflick limit, which restricts their proliferative capacity to their telomere length, shortened in every replication [5, 29, 30]. However, cells subjected to continuous stress show similar phenotypes to cells with replicative senescence in a matter of hours to days, independent of telomere length, a phenomenon called stress-induced premature senescence or accelerated senescence [3, 5]. Oxidative stress and pro-inflammatory mediators, such as the SASP-related cytokines, can act as cell stressors leading to premature paracrine cellular senescence [31-33]. In particular, ROS, interferon (IFN)-α, tumor necrosis factor (TNF)-α, and prostaglandin (PG)E_2_ can trigger the loss of CD28 expression and induce the formation of CD28^-^ T lymphocytes with senescent characteristics [34-38].

**Figure 3.**
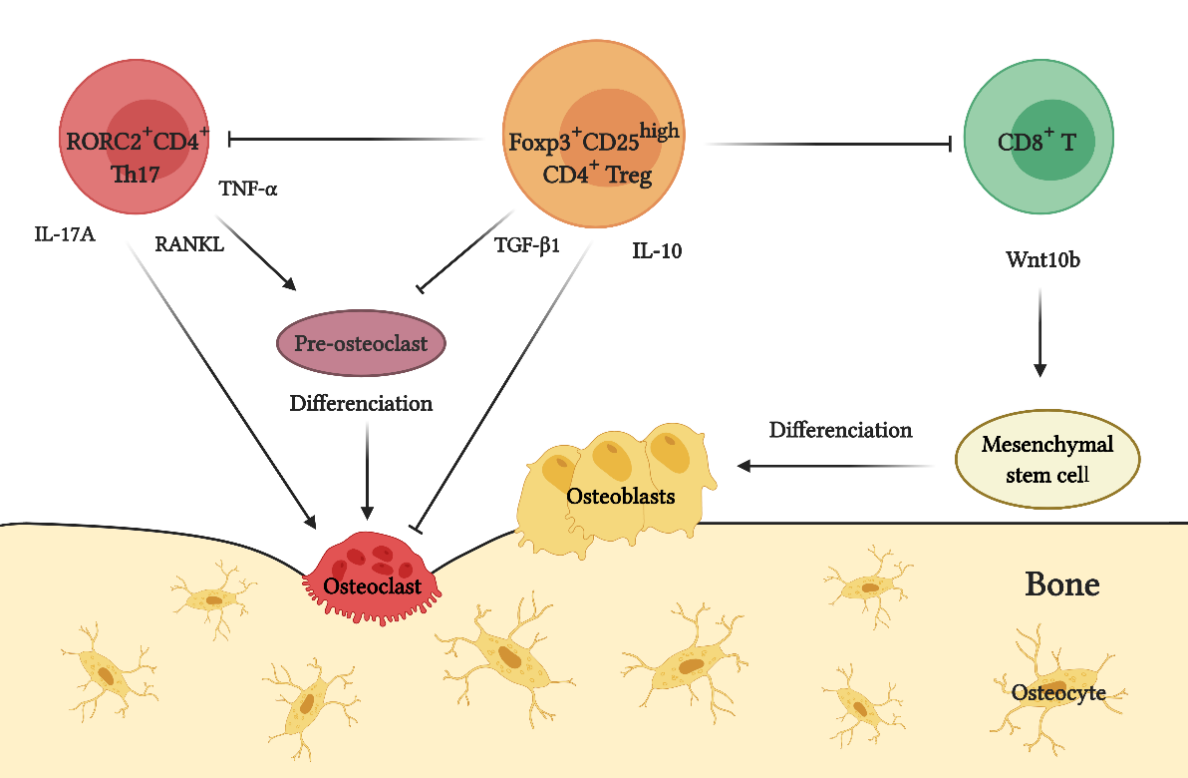
Role of T lymphocytes during osteoimmunology. Under physiological conditions, the balance between the osteolytic activity of T-helper type-17 (Th17) lymphocytes and bone protective activity of T regulatory (Treg) lymphocytes controls the maintenance of bone tissue homeostasis. Th17 lymphocytes express the transcription factor retinoid acid receptor-related orphan nuclear receptor C2 (RORC2) and induce osteoclastogenesis and activation of mature osteoclasts by producing tumor necrosis factor (TNF)-α, interleukin (IL)-17A, and receptor-activator of nuclear factor κB ligand (RANKL). Treg lymphocytes express the transcription factor forkhead box P3 (Foxp3), overexpress the cell surface marker CD25, and inhibit osteoclastogenesis and osteoclast activity mainly by producing transforming growth factor (TGF)-β1 and IL-10. It is noteworthy that Foxp3^+^CD25^high^ Treg lymphocytes can directly inhibit the differentiation, proliferation, and function of RORC2^+^ Th17 lymphocytes. Moreover, Foxp3^+^CD25^high^ Treg lymphocytes can promote the production of Wnt10b by CD8^+^ T lymphocytes, which induces the differentiation of osteoblasts precursors through the Wnt-β-catenin-signaling pathway and consequently promotes bone formation (Created with BioRender.com).

While CD28 is expressed in most murine T lymphocytes, CD28 is expressed in 90% of CD4^+^ T lymphocytes and only in 50% of CD8^+^ T lymphocytes in humans; thus, the loss of CD28 expression seems to be a good senescence marker for CD4^+^ T lymphocytes but less specific for CD8^+^ T lymphocytes [15]. In this context, the expression of CD27, another costimulatory receptor constitutively expressed in T lymphocytes, has also been used to differentially identify senescent CD4^+^ and CD8^+^ T lymphocytes. Indeed, senescent CD8^+^ T lymphocytes first lose the CD28 expression and, in a later stage, they lose the CD27 expression, while CD4^+^ T lymphocytes lose first the CD27 expression and then the CD28 expression [26]. Both CD4^+^CD27^-^CD28^-^ and CD8^+^CD27^-^CD28^-^ T lymphocytes downregulate the expression of molecules specifically involved in the TCR signaling pathway, such as Zap70 and Lck [18, 39]. Interestingly, downregulation of these signalosome components does not imply a state of anergy or cellular exhaustion. In fact, senescent T lymphocytes can retrieve the expression of CD45RA and express other signaling molecules that contribute to the maintenance of cell functionality, such as CD11b, CD57, and killer cell lectin-like receptor subfamily G member 1 (KLRG1) [40-42]. Therefore, all these markers can be used to identify the different subsets of senescent T lymphocytes (Fig. 2). Recent reports reveal that, in CD8^+^CD27^-^CD28^-^ T lymphocytes, sestrins induce natural killer functions [39], while in CD4^+^CD27^-^CD28^-^ T lymphocytes, sestrins induce phosphorylation of distinct mitogen-activated protein kinases (MAPKs) [18]. Thus, MAPKs activation mediates different aspects of CD4^+^ T-cell senescence; for instance, the activity of p38-MAPK has been associated with low telomerase activity, ERK with the activation of DDR and upregulation of γ-H2Ax, and JNK with the signalosome inhibition [18].

## Osteoimmunology: Role of T lymphocytes

Osteoimmunology studies the relation and cross-communication between the immune and bone systems. Although osteoimmunology initially focused on the influence of the immune response on osteoclast activity, the key cell responsible for bone resorption, nowadays it also involves its effects over osteoblast activity and bone formation [43, 44]. Hence, the immune response is closely related to the maintenance of bone homeostasis, which ultimately depends on the molecularly regulated cellular coupling between osteoclasts and osteoblasts (Fig. 3) [43, 45].

Osteoimmunological control of osteoclast function comprises the activity of a triad of proteins belonging to the superfamily of TNF ligands and receptors, which are the receptor-activator of nuclear factor κB ligand (RANKL), its functional receptor (RANK), and its soluble decoy receptor osteoprotegerin (OPG) [46]. In general terms, RANKL induces osteoclast differentiation and activation by interacting with its specific receptor RANK, expressed in osteoclast precursors and mature osteoclasts; otherwise, OPG inhibits the RANKL/RANK interaction and arrests osteoclastogenesis and bone loss [46]. The RANKL/RANK signaling induces the activation of the transcription factors termed nuclear factor kappa-light-chain-enhancer of activated B cells (NFκB) and nuclear factor of activated T cells-cytoplasmic 1 (NFATc1), considered as the master regulators during osteoclastogenesis [43]. Activation of NFκB and NFATc1 subsequently induces the expression of the osteoclast-characteristic genes implied in the process of bone resorption, including dendritic cell-specific transmembrane protein (DC-STAMP), cathepsin-K, and tartrate-resistant acid phosphatase (TRAP) [43]. Although infrequently, RANKL-independent osteoclast differentiation mechanisms have also been described, such as those dependent on TNF-α or interleukin (IL)-17A signaling. In experimental conditions, the combination of TNF-α and macrophage colony-stimulating factor (M-CSF) induces functional TRAP^+^ osteoclasts, and this induction can be completely blocked by using neutralizing antibodies against the TNF-α receptor instead of OPG or RANK neutralizing antibodies [47, 48]. Similarly, IL-17A combined with M-CSF triggers the formation of TRAP^+^ cathepsin-K^+^ DC-STAMP^+^ osteoclasts in the absence of exogenous RANKL [49, 50].

At a cellular level, the osteoimmunological regulation of osteoclasts development and activity is mediated by T lymphocytes, particularly T-helper type-17 (Th17) and T regulatory (Treg) lymphocytes, which have opposite roles during immune surveillance, autoimmunity, and inflammation [46, 51]. Th17 lymphocytes express the transcription factor retinoid acid receptor-related orphan nuclear receptor C2 (RORC2), the master-switch gene that determines their differentiation and pro-inflammatory functions [52, 53]. On the other hand, Treg lymphocytes express the transcription factor forkhead box P3 (Foxp3), the master-switch gene that determines their differentiation and regulatory functions, by producing anti-inflammatory cytokines, such as transforming growth factor (TGF)-β1 and IL-10 [51, 54]. Th17 cell pro-inflammatory role has been implicated in many chronic inflammatory and osteolytic diseases, while Treg cell anti-inflammatory role has been associated with the maintenance of immune tolerance and regulation of effector T lymphocyte activity, including Th17 lymphocytes [46, 51]. Under physiological conditions, the balance between the activity of Th17 and Treg lymphocytes allows the dynamic regulation of bone homeostasis. However, chronic inflammation induces a Th17/Treg imbalance, characterized by the increased activity of IL-6, IL-17A, and RANKL-producing Th17 lymphocytes and phenotypically unstable Treg lymphocytes, which lose their regulatory capacity [51, 55]. In addition, this inflammatory milieu further favors the production of pro-inflammatory cytokines, including IFN-γ, TNF-α, and IL-1β, which can induce the expression of RANKL in osteoblasts and fibroblasts with osteoclastogenic capacity, thus generating a complex network of RANKL-producing cells that contribute even more to pathologic bone loss [46].

Otherwise, osteoimmunological control of osteoblast function and bone formation depends on the Wnt signaling pathway. In osteoblasts precursors such as mesenchymal stromal cells, the binding of Wnt10b to its coreceptor, presumably low-density lipoprotein receptor-related protein 5 (LRP5), leads to β-catenin nuclear translocation, which in turn induces the expression of the transcription factors responsible for osteoblastogenesis and necessary for bone formation, such as runt-related transcription factor 2 (Runx2), distal-less homeobox 5 (Dlx5), and osterix (Osx) [56]. At a cellular level, the osteoimmunology of osteoblast function relies on the interaction between Treg and CD8^+^ T lymphocytes. Indeed, the increase in the number and activity of TGF-β1-producing Treg lymphocytes drives the assembly of the NFAT1/SMAD3 transcription complex in CD8^+^ T lymphocytes and Wnt10b production, and consequently, osteoblast differentiation and bone formation [44, 57, 58].

**Figure 4.**
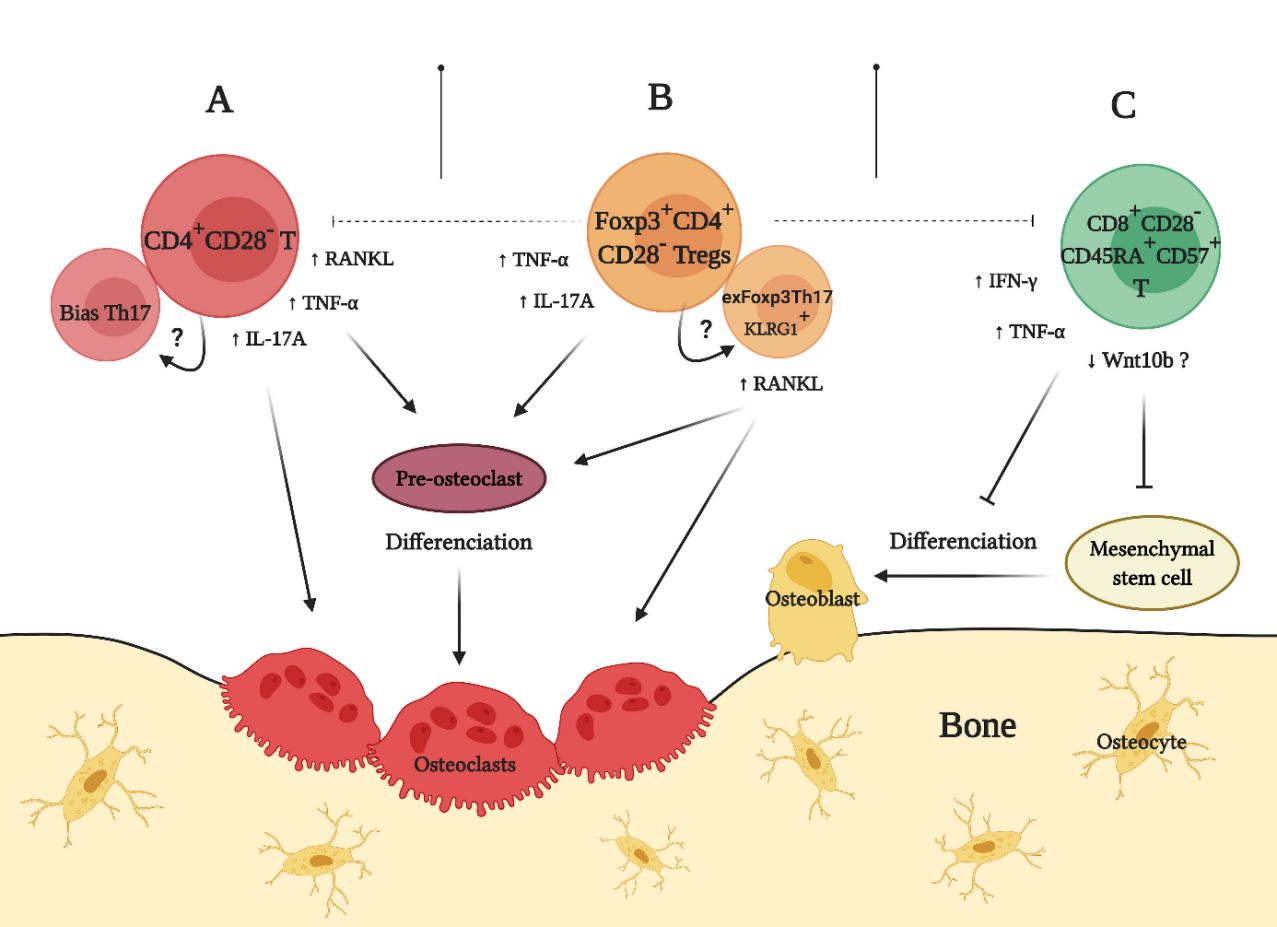
Role of premature senescent T lymphocytes during pathological bone loss. (A) Premature senescent CD4^+^CD28^-^ T lymphocytes promote osteoclastogenesis and osteolysis by producing increased levels of tumor necrosis factor (TNF)-α, interleukin (IL)-17A, and receptor-activator of nuclear factor κB ligand (RANKL). (B) Premature senescent Foxp3^+^CD28^-^ Treg lymphocytes show phenotypic instability, downregulated CD25 expression, and decreased immunosuppressive capacity. In consequence, these Treg lymphocytes could lose Foxp3 expression and trans-differentiate into exFoxp3Th17 cells, which express killer cell lectin-like receptor G1 (KLRG1) and acquire osteoclastogenic and bone resorptive capacities by producing TNF-α, IL-17A, and RANKL. (C) Premature senescent CD8^+^CD28^-^CD45RA^+^CD57^+^ T lymphocytes inhibit osteoblastogenesis and bone formation by producing TNF-α and interferon (INF)-γ. In addition, these senescent CD8^+^ T lymphocytes downregulate their Wnt10b production and consequently affect the Wnt-β-catenin-dependent osteoblastogenesis and bone formation; however, this mechanism has not been entirely demonstrated yet (Created with BioRender.com).

## Premature senescence of T lymphocytes and pathological bone loss

Despite having different etiologies, chronic diseases characterized by manifesting pathological bone loss usually share osteoimmunological similarities that affect the target tissues. In some of these diseases, premature senescence of T lymphocytes has been related to increased Th17 lymphocyte activity and RANKL production, which induces osteoclast differentiation and provokes adverse effects on bone structure and function (Fig. 4A). In fact, finding an increased number of prematurely senescent CD4^+^CD28^-^ T lymphocytes has become relatively common in rheumatoid arthritis, juvenile idiopathic arthritis, ankylosing spondylitis, osteopenia, osteoporosis, and osteomyelitis [59-64]. Apart from that, CD4^+^CD28^-^ T cells have also been reported in HIV-infected individuals and lupus erythematosus-affected patients, in which their activity could explain the increased risk of developing osteoporosis observed in these diseases [65-68].

In the case of rheumatoid arthritis, CD4^+^CD28^-^ T lymphocytes produced higher levels of RANKL as compared with CD4^+^CD28^+^ T lymphocytes, and these senescent CD4^+^CD28^-^ T lymphocytes showed a greater capacity to induce the differentiation of TRAP^+^ osteoclasts and bone resorption when compared with non-senescent CD4^+^CD28^+^ T lymphocytes [62]. Also, CD4^+^CD28^-^ T lymphocytes isolated from the synovial fluid of rheumatoid arthritis-affected patients were capable of producing higher levels of IL-17A as compared with isolated CD4^+^CD28^+^ T lymphocytes [69, 70]. During osteoporosis, CD4^+^CD28^-^ T lymphocytes expressed higher levels of TNF-α and intensely induced the activation of TRAP in osteoclasts as compared with CD4^+^CD28^+^ T lymphocytes [71]. Besides, in an animal model of osteoporosis induced by ovariectomy, when the immunoprotective effect of the neutralizing antibodies anti-RANKL, anti-TNF-α, or anti-IL-17A was compared, IL-17A blockade led to a more potent reduction in the number of CD4^+^CD28^-^ T lymphocytes as compared with RANKL or TNF-α blockade, which translated into an improvement in skeletal parameters and demonstrated the key role for IL-17A in the CD4^+^ T-cell senescence [72]. During osteomyelitis, an increased number of CD4^+^CD28^-^ T lymphocytes was observed in bone tissue, which was associated with long-term activated T cells, CD11b overexpression, and enhanced cytotoxic ability, all characteristics of T-cell senescence [64, 73]. Although the presence and activity of CD4^+^CD28^-^ T lymphocytes during periodontitis, the most prevalent osteolytic disease in humans, has not been reported, the continuous inflammatory signals and the persistent antigenic stimulus, provided by microbial dysbiosis and the destruction of periodontal tissues, could trigger the loss of CD28 expression and senescence of CD4^+^ T lymphocytes [74, 75].

Similar to effector CD4^+^ and CD8^+^ T lymphocytes, Treg lymphocytes can also lose the CD28 expression and acquire a senescent phenotype and related functions (Fig. 4B). In rheumatoid arthritis-affected patients, an increase in the number of Foxp3-expressing CD4^+^CD28^-^ T lymphocytes has been reported, and these senescent Treg cells expressed lower levels of CD25, showed higher SA-βgal activity, and produced higher levels of TNF-α, IFN-γ, and IL-17A, as compared with Foxp3^+^CD4^+^CD28^+^ lymphocytes [16]. Interestingly, no differences in telomere length were observed in these Foxp3^+^CD4^+^CD28^-^ lymphocytes, suggesting a state of premature senescence rather than replicative senescence [16].

In rheumatoid arthritis and periodontitis, the presence of unstable Treg lymphocytes that have lost the expression of Foxp3 and their regulatory capacities have been described [76, 77]. These lymphocytes, termed exFoxp3Th17 lymphocytes, acquire the capacity to produce a Th17-pattern of cytokines, such as IL-17A and RANKL, and even show a greater osteoclastogenic capacity than regular Th17 lymphocytes [76, 77]. Interestingly, exFoxp3Th17 lymphocytes, apart from presenting the loss of Foxp3 and downregulated CD25 expression, upregulate the expression of the senescence marker KLRG1 [76, 77]. In fact, KLRG1^+^ Treg lymphocytes show low proliferative capacity and impaired suppressive functions *in vitro* and *in vivo*, frequently losing the Foxp3 expression and reprogramming their activity towards a Th17 effector profile [78]. In this context, exFoxp3Th17 lymphocytes could correspond, at least in part, to senescent Treg cells that have lost their CD28 expression, which also has a direct role in the Foxp3 expression and the maintenance of high levels of CD25 expression [79, 80]. Thus, exFoxp3Th17 lymphocytes could lose the ability to regulate Foxp3 and CD25 through the absence of CD28 co-stimulation, favoring the Th17/Treg imbalance, increasing the production of SASP-related cytokines, and inducing osteoclastogenesis and pathological bone resorption.

## Premature senescence of T lymphocytes and bone formation

The senescence of CD8^+^ T lymphocytes has been negatively associated with bone formation and regeneration (Fig. 4C). Senescent CD8^+^CD28^-^CD45RA^+^ CD57^+^ T lymphocytes inhibit osteoblast differentiation and human mesenchymal stromal cell survival, leading to a fewer number of osteoblast precursors [81]. In addition, CD8^+^CD28^-^CD45RA^+^CD57^+^ T lymphocytes have been identified as the main producers of pro-inflammatory cytokines, such as TNF-α and IFN-γ, during bone fracture healing failure [81]. Indeed, TNF-α and IFN-γ seem to be part of the SASP in these senescent T lymphocytes [82]. Furthermore, in aged mice, the number and activity of CD8^+^CD28^+^ T lymphocytes decreases and associates with the downregulation of Wnt production [83]; however, it is unknown whether the production of Wnt10b by premature senescent CD8^+^CD28^-^CD45RA^+^CD57^+^ T lymphocytes is altered, which could also affect the process of bone formation. Additionally, CD8^+^ T lymphocytes must be in an anergic state to produce Wnt10b [84]; thus, senescent Treg lymphocytes with impaired suppressive functions could be unable to induce CD8^+^ T lymphocytes to an anergic state, affecting osteoblastogenesis and bone formation. Indeed, animal models with deficient Treg function show reduced proliferation, differentiation, and lifespan of osteoblasts, and consequently, less bone formation mediated by CD8^+^ T lymphocytes [58].

## SASP in senescent T lymphocytes

Phosphorylation of p38-MAPK is increased in both senescent CD8^+^ and CD4^+^ T lymphocytes [82, 85]. In senescent CD8^+^ T lymphocytes, p38-MAPK signaling governs SASP and regulates the production of SASP-related cytokines [82, 86]. In addition, p38-MAPK inhibits autophagy independently of mTOR, by keeping the p38-MAPK interacting protein (p38IP) sequestered, which is necessary to translocate the autophagy-related protein (ATG)-9 to the autophagosome [82]. These changes lead to the accumulation of dysfunctional mitochondria and increased amounts of ROS, which favor the production of SASP-related pro-inflammatory cytokines, such as TNF-α and IFN-γ, contributing to the inhibition of bone formation [81, 82].

In senescent CD4^+^ T lymphocytes, the constitutive phosphorylation of p38-MAPK is determined by a sestrin-mediated intrasensory signaling pathway dependent on the DNA damage [18, 85, 87]. In these cells, it is possible that p38-MAPK signaling can also govern SASP; however, this phenomenon has not been described yet. In elderly people, defective autophagy, accumulation of dysfunctional mitochondria, and increased ROS production in CD4^+^ T lymphocytes leads to intracellular STAT3 phosphorylation and Th17-type cytokine production [88]. All these mechanisms are dependent on p38-MAPK phosphorylation, and additionally, SASP-related cytokines produced by CD4^+^CD28^-^ T lymphocytes, including TNF-α, IL-17A, and RANKL, could also contribute to osteoclastogenesis and bone loss.

**Figure 5.**
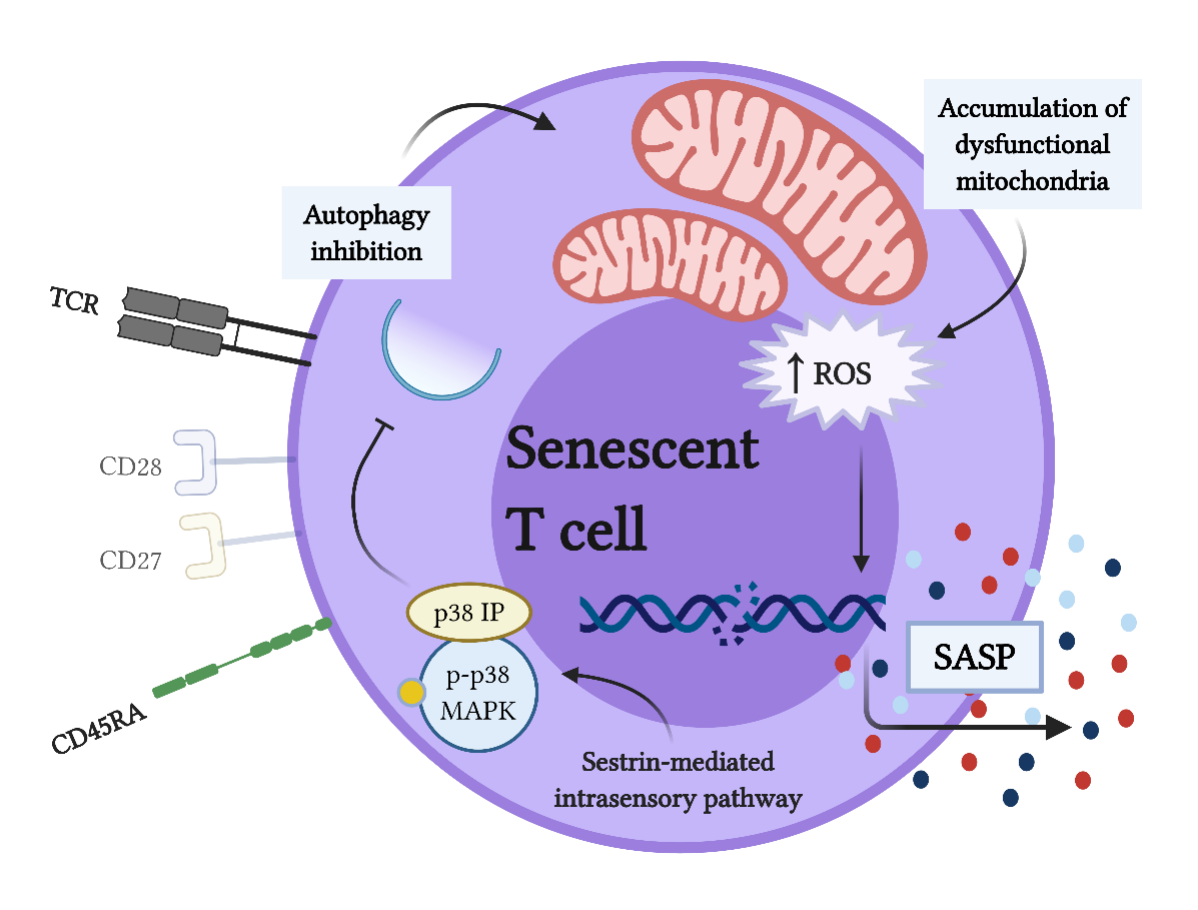
Phosphorylation of p38-MAPK drives SASP-related cytokine production in premature senescent T lymphocytes. Senescent T lymphocytes exhibit phosphorylation of p38-mitogen-activated protein kinase (p38-MAPK) by a sestrin-mediated intrasensory signaling pathway that is maintained over time. Phosphorylation of p38-MAPK (p-p38-MAPK) inhibits autophagy by sequestering the p38-MAPK interacting protein (p38IP), necessary to translocate autophagy-related protein (ATG)-9 to the autophagosome. Inhibition of autophagy results in defective clearance of dysfunctional mitochondria, which accumulate and cause the rise of intracellular reactive oxygen species (ROS) levels that act as inducers of the senescence-associated secretory phenotype (SASP)-related cytokine production (Created with BioRender.com).

Taken together, the p38-MAPK phosphorylation could be a conserved mechanism in senescent CD8^+^ and CD4^+^ T lymphocytes that contributes to the explaining of the upregulation of SASP-related pro-inflammatory and pro-osteoclastogenic mediators, including RANKL, triggered by the inhibition of autophagy, accumulation of dysfunctional mitochondria, and increased ROS production (Fig. 5).

## Therapeutic strategies aimed at controlling premature senescence of T lymphocytes

Nowadays, different therapeutic strategies have been proposed to specifically target cellular senescence. These emerging senotherapeutic approaches comprise senolytic strategies, which seek to eliminate senescent cells, and senomorphic strategies, which can partially reverse the senescent phenotype, including the establishment of SASP [89]. Although senolytic strategies have been reported to be very beneficial and do not seem to have negative long-term consequences, this approach has not been evaluated in senescent T lymphocytes [89]. On the contrary, different senomorphic strategies have been evaluated in senescent T cells using diverse *in vitro* and *in vivo* models, including metformin, sestrin blockade, p38-MAPK inhibitors, and TNF-α1-receptor blockade [18, 37, 39, 82, 88, 90].

In CD4^+^ T lymphocytes obtained from elderly people, metformin rescued autophagy and mitochondrial function, inhibited the production of ROS, and downregulated the production of Th17-associated cytokines, IL-6, IL-17A, and IL-21 [88]. In addition, blocking sestrins in senescent CD4^+^ T lymphocytes reduced the damaged DNA accumulation and enhanced the cell proliferative potential and telomerase activity, while its blockade in senescent CD8^+^ T lymphocytes negatively regulated natural killer functions [18, 39]. Similarly, inhibition of p38-MAPK signaling in senescent CD4^+^ T lymphocytes significantly improved telomerase activity and cell survival after TCR activation, while its inhibition in senescent CD8^+^ T lymphocytes increased autophagic activity, restored mitochondrial function, and downregulated the TNF-α expression [82, 90]. Finally, blocking TNF-α1-receptor in senescent CD8^+^ T lymphocytes increased their proliferative potential, delayed the loss of CD28 expression, and improved telomerase activity [37]. However, to our knowledge, none of these senomorphic strategies have been evaluated in premature senescent T lymphocytes; thus, making the analysis of senotherapeutic approaches for osteolytic diseases, in the context of immunosenescence, novel and attractive.

## Conclusion

With age, the inherent cellular mediated immunity flaws related to inflamm-aging increase the vulnerability of the immune system, which can lead to defective bone homeostasis. In addition, senescent T lymphocytes can also be generated prematurely during chronic inflammatory diseases regardless of chronological aging. In the context of osteoimmunology, the activity of T lymphocytes with senescent phenotype contributes to a novel understanding of the cross-communication between the immune response and bone metabolism. During osteolytic diseases, premature senescence of T cells can be crucially involved in the increased activity of RANKL-producing Th17 and exFoxp3Th17 lymphocytes, which cause the Th17/Treg imbalance and bone loss. These new findings uncover the need for the exploration of innovative therapeutic strategies focused on the control of immunosenescence related pathological bone loss. In fact, considering that the loss of CD28 expression and the SASP-related RANKL production are distinctive features of premature senescent T lymphocytes during osteolytic diseases, these could be a focus of therapeutic attention since these changes could potentially be reversible.
